# Comparison of two spectrometric counting modes for fast analysis of selected radionuclides activity

**DOI:** 10.1007/s10967-015-4688-y

**Published:** 2016-01-11

**Authors:** Magdalena Długosz-Lisiecka

**Affiliations:** Technical University of Lodz, Institute of Applied Radiation Chemistry, Wróblewskiego 15, 90-924 Łódź, Poland

**Keywords:** Anticoincidence mode, Coincidence photons, Activity equilibrium

## Abstract

The two counting modes: a normal with a single HPGE detector and second with the additional anti-Compton shield of the annular NaI(Tl) detector have been compared for fast determination of the activity concentration of thorium ^232^Th in the building materials. The ^232^Th activity concentration was calculated by measurement of its decay products: ^212^Pb, ^212^Bi and ^208^Tl as well ^228^Ac content. Although the Compton suppression mode applied in gamma spectrometry systems in general increase sensitivity of the analysis, but in case of 583 keV the most abundant ^208^Tl *γ*-line, the significant reduction of photon counting rate was observed.

## Introduction

Natural thorium radionuclides are always present in environmental samples, e.g. in most rocks and soils, thorium is about three times more abundant than uranium [1]. Soil commonly contains an average of around six parts per million (ppm) of thorium. Mineral waste byproducts and different kinds of building materials can contain high elevated levels of thorium radionuclides as well. According to the EU Directive [2] regarding basic safety standards for protection against the dangers arising from exposure to ionizing radiation, for the purposes of identifying types of building materials of concern from a radiation protection point of view, the activity concentrations of primordial radionuclides ^226^Ra, ^232^Th (or its decay product ^228^Ra) and ^40^K should be determined. The activity concentration index I is given by the following formula:

1 $$I = \frac{{{\text{C}}_{{{\text{Ra}}226}} }}{300} + \frac{{{\text{C}}_{{{\text{Th}}232}} }}{200} + \frac{{{\text{C}}_{{{\text{K}}40}} }}{3000}$$

where C_Ra226_, C_Th232_ and C_K40_ are the activity concentrations in Bq/kg in the building material and the numbers 300, 200 and 3000 are the weighting factors of the corresponding radionuclides, respectively [3–5].

Natural radioactivity in solid environmental samples, including building materials, can be routinely measured by gamma spectrometry system based on various types of detectors. Unfortunately, both long-lived natural thorium radionuclides—^232^Th and ^228^Th—are almost pure *α* emitters with very low *γ* emission rates: 63.8 keV—(0.267 %) and 84 keV—(1.22 %). However, because relatively shorter half-lives of all thorium daughters in the majority of environmental solid samples the radioactive equilibrium for all members of the thorium chain is usually achieved.

If measurement of natural radioactivity in solid environmental samples is provided by a single HPGe or NaI detector, one should take into account the problem known as coincidence summing correction. Usually this problem can be routinely solved for each peak energy line by using special software, e.g. LABSOCS (model S574 LabSOCS Calibration Software, Canberra). Coincidence summing correction is important for isotopes emitting two or more photons in a cascade, e.g. ^60^Co [6].

Developed anticoincidence (AC) system with a Compton suppression shield has been integrated with a primary HPGe detector and used for measuring low level activity in environmental samples [7, 8]. Due to Compton scattering, only part of initial photon energy is absorbed within the active volume of the germanium counter.

A scattered photon can escape from this detector and can be detected at the same time in the surrounding scintillation counter. In a modern active Compton suppression shield with an HPGe as the primary detector, the problem of two or more photon coincidence can occur. This can be a serious problem, because the large shielding NaI(Tl) and HPGe detectors are working in AC mode to lower the Compton background [9–11]. Therefore, the two photons coming in cascade from the same nuclide decay may be simultaneously detected by both counters and consequently eliminated, lowering the counting efficiency for that particular *γ* line. Therefore, although the AC system AC mode reduces the natural background of the measurement, it can also reduce signals that occur at the same time interval on both detectors.

**Fig. 1:**
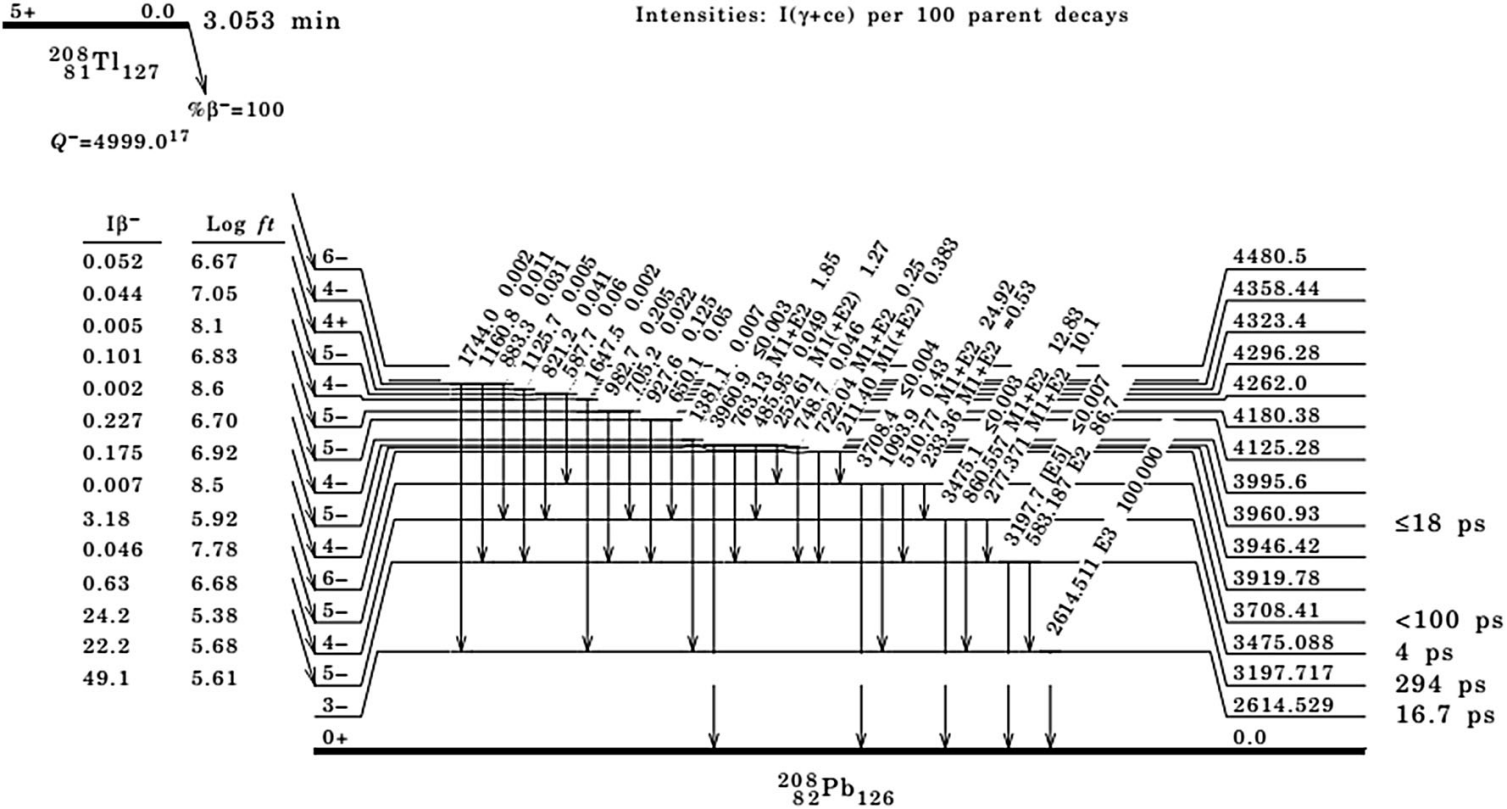
^208^Tl decay scheme [14]

In routine measurements, the time range of the AC mode is equal to 5 µs, and multi-detector systems are synchronized by the time and energy of the events. To extract coincident events in AC mode, complex electronics are often used to identify accidental coincidences during measurement. As it is evident from Fig. 1. ^208^Tl radionuclide decays with series of cascade emitting photons with interval times in order of pico or nanoseconds are much shorter than AC gate width interval equal mostly about 5 µs. Therefore, majority of them can be rejected from the AC spectrum. In single HPGe mode of system such rejection is based only on coincidence summing correction phenomenon, which seems to be negligible for ^232^Th daughters. Therefore figure of merit (FOM) parameter has been used to characterize the performance of both modes of measurements to determine their relative utility for an application. The objective of the present study is to choose the proper counting mode for ^232^Th radionuclide progeny based on the FOM criterion.

## Materials and methods

Several types of environmental sample as well as building material (four types of brick, three types of tile, two types of ceramic) were collected from different local ceramic producers in Poland. The samples were crushed to a fine powder then dried at 100 °C for 24 h to a constant weight. The geometry of the samples was normalized to disc form with a diameter of 51 mm and height of 10 mm. The samples were sealed and after one week delay, measured using the analytical AC mode at 80,000 s.

For measuring natural radioactivity, low-level gamma spectrometry with an active and passive suppression system was used. An HPGe GX3020 counter, with relative efficiency equal to 30 % and 2002CLS (Canberra Inc.) offset preamplifier, was used as the primary detector. The anti-Compton veto system manufactured by SCIONIX (The Netherlands), consisting of a scintillating NaI(Tl) crystal in aluminum housing with six 3′ photo-multiplier-tubes to collect the scintillation light and one for the end cap was used as a secondary detector. Both detectors were integrated in a massive low activity lead house and the interior of the lead house was filled with pure nitrogen gas that evaporating from a Dewar vessel.

The accuracy of the method was checked by measuring under the same geometry as the samples, the two reference materials: IAEA Soil 327 and Soil 375.

## Results

^232^Th radionuclide can be analyzed by almost any progeny isotope present in its decay chain. In routine *γ* spectrometry measurement, ^228^Ac and/or ^212^Bi and/or ^208^Tl and/or ^212^Pb radionuclides are often chosen for this analysis [4, 5, 12]. However, one should remember that in the case of ^208^Tl, only 35.94 % of ^212^Bi decay leads to this radionuclide, while 100 % of ^228^Ac or ^212^Pb activity are in real secular equilibrium with ^232^Th.

Activity concentration for all chosen radionuclides was calculated on the basis of Eqs. (2) and (3):

2 $$A_{x} = \frac{{I_{x} - I_{BKG\left( x \right)} }}{{\varepsilon_{\left( x \right)} m }}$$

3 $$\varepsilon_{\left( x \right)} = \varepsilon_{\det \left( x \right)} \varepsilon_{\gamma \left( x \right)} \varepsilon_{red\left( x \right)}$$

where $A_{x}$—activity concentration of x-isotope [Bq/kg], $I_{x}$—count rate for *x*-isotope in the region of the chosen photon energy line for the sample [imp/sec], $I_{BKG\left( x \right)}$—count rate for *x*-isotope in the region of the chosen photon energy line for the background [imp/sec], $m$—mass of the sample [kg], $\varepsilon_{\left( x \right)}$—absolute efficiency for the chosen photon energy line, $\varepsilon_{{{ \det }\left( x \right)}}$—detection efficiency for the chosen photon energy line estimated on the basis of LABSOCS software, $\varepsilon_{\gamma \left( x \right)}$—intensity of the gamma-ray emission, $\varepsilon_{red\left( x \right)}$—AC reduction factor calculated as ratio of count rate for the chosen photon energy line in AC mode in relation to the count rate based on a single HPGe measurement.

^232^Th activity concentrations were determined simultaneously by the progenies ^212^Pb (238 keV *γ* energy line), ^212^Bi (727 keV), ^208^Tl (583 or 860 keV) and ^228^Ac (911.1 keV). All these radionuclides emit relatively high-energy *γ* photons with energies >200 keV, and therefore self-absorption correction in small thickness samples (<5 mm) is not necessary.

The AC counting mode can substantially reduce the count rate of the photons emitted in cascade decay, particularly for the most abundant (84.5 %) 583 keV photons of ^208^Tl (Fig. 1). In that case, practically at the same time (which is shorter than settled AC intervals), photons with energies 277, 727, 511 and 2614 keV are simultaneously emitted [13]. The high-efficiency *γ*–*γ* coincidence events rejected from the AC spectrum can significantly disturb regular spectrometric measurements and as a result reduce the activity concentration of analyzed radionuclides.

In order to choose the proper *γ* line of the examined radionuclides and the best counting mode, the FOM criterion was calculated. The FOM is commonly defined as in Eq. (4):

4 $${\text{FOM}} = \frac{{\varepsilon_{(x)}^{2} }}{{I{}_{BKG(x)}}}$$

The FOM was calculated for each energy peak of ^212^Pb, ^212^Bi, ^208^Tl and ^228^Ac for normal and AC counting modes for two reference materials: IAEA soil 327 and IAEA soil 375 [15, 16].

Using AC shield has influence on the count rate of various peak implicit on their detection efficiency *ε*_(*x*)_. The absolute efficiency *ε*_(*x*)_ of the detection system was determined through a series of measurements using two of reference materials obtained from the IAEA; soil 327 and soil 375 (Table 1).

**Table 1:** Efficiency and activity of ^212^Pb, ^212^Bi, ^208^Tl and ^228^Ac in secular equilibrium with ^232^Th in reference materials

Isotope	*γ* energy line [keV]*	$\varepsilon_{\gamma \left( x \right)}$ [%]*	$\varepsilon_{{{ \det }\left( x \right)}}$	$\varepsilon_{red\left( x \right)}$	$\varepsilon_{\left( x \right)AC}$	FOM_AC_	$\varepsilon_{(x)HPGe}$	FOM _HPGe_	Measured activity concentration of ^232^Th [Bq/kg]	Certified activity concentration of ^232^Th [Bq/kg]	Reference material
					Anticoincidence		single HPGe				
^212^Pb	238.57	43.6	0.1149	1.000	0.05010	0.5070	0.05010	0.5486	38.6 ± 2.2	38.7	IAEA soil 327
^208^Tl	583.02	86.0	0.0477	0.221	0.00907	0.0967	0.04102	0.7238	38.1 ± 1.8	38.7	
	860.3	12.0	0.0316	0.282	0.00107	0.0041	0.00379	0.0521	37.9 ± 2.3	38.7	
^228^Ac	911.16	29.0	0.0319	0.821	0.00760	0.2006	0.00925	0.1141	38.5 ± 2.0	38.7	
^212^Bi	727.25	6.65	0.0390	0.786	0.00204	0.0115	0.00259	0.0174	37.5 ± 2.6	38.7	
^212^Pb	238.57	43.6	0.1154	0.997	0.05016	0.5084	0.05031	0.5533	20.6 ± 1.4	20.5	IAEA soil 357
^208^Tl	583.02	86.0	0.0483	0.208	0.00864	0.0878	0.04154	0.7421	19.9 ± 1.1	20.5	
	860.3	12.0	0.0319	0.310	0.00119	0.0056	0.00382	0.0532	20.3 ± 1.6	20.5	
^228^Ac	911.16	29.0	0.0326	0.812	0.00768	0.2050	0.00945	0.1192	19.8 ± 1.5	20.5	
^212^Bi	727.25	6.65	0.0392	0.794	0.00207	0.0118	0.00261	0.0175	21.4 ± 1.7	20.5	

* Data taken from [17]

Table 1 shows that the highest absolute efficiency not reduced by the AC mode was for 238 keV for the ^212^Pb isotope. In this counting mode FOM has the highest value equal average 0.5486. In measurement with using single HPGe detector the highest FOM has ^208^Tl for 583 keV peak energy line [17]. Based on this, it can be concluded that the activity concentration of ^232^Th can be measured with the highest precision using this line of the ^208^Tl isotope.

The results of measuring the activity concentration of ^232^Th in the examined samples, calculated as average activities of its progenies are presented in Table 2.

**Table 2:** Measured activity of the ceramic materials in AC mode and with efficiency reduction correction

Type of sample	Mass [g]	A_Pb-212_ [Bq/kg]	A_Bi-212_ [Bq/kg]	A_Ac-228_ [Bq/kg]	A_Tl-208_ [Bq/kg]	A_Th-232_ [Bq/kg]
Chinese plate	45.6	104.2 ± 6.7	76.3 ± 3.2 (97.3)	79.9 ± 4.9 (97.7)	23.3 ± 1.8 (105.4)	101.1
Italian plate	29.9	41.1 ± 2.2	33.6 ± 2.5 (42.8)	34.5 ± 2.6 (42.0)	8.55 ± 0.93 (39.5)	41.5
Wall part	33.9	41.5 ± 2.1	32.6 ± 2.4 (41.5)	36.7 ± 2.5 (41.7)	9.02 ± 0.84 (40.85)	41.4
Polished tile	41.4	42.2 ± 2.3	34.3 ± 2.9 (43.7)	35.9 ± 2.8 (43.9)	8.87 ± 0.85 (40.6)	42.8
Ceramic plate	42.6	44.5 ± 2.2	32.3 ± 2.5 (41.2)	34.2 ± 2.9 (41.8)	9.07 ± 0.75 (41.1)	42.1
Chimney block	30.2	32.0 ± 1.8	25.7 ± 2.7 (32.8)	25.9 ± 2.2 (31.6)	7.23 ± 0.77 (33.2)	32.4
Unbaked brick	39.0	21.5 ± 1.8	19.9 ± 1.7 (25.4)	18.8 ± 1.4 (22.9)	5.25 ± 0.52 (23.7)	23.4
Chinese cup	43.7	50.2 ± 3.2	39.2 ± 2.7 (50.1)	43.1 ± 1.4 (52.5)	10.89 ± 0.81 (49.3)	50.5
Ceramic plate (brown)	30.8	39.2 ± 2.0	33.1 ± 2.5 (42.2)	33.7 ± 1.6 (41.1)	8.97 ± 0.85 (40.6)	40.8
Strzegom granite	30.5	56.7 ± 5.7	43.4 ± 3.2 (55.3)	44.2 ± 3.8 (53.9)	11.5 ± 0.9 (52.1)	54.5
Vesuvius lava	30.6	42.1 ± 2.8	33.9 ± 6.3 (43.1)	32.1 ± 2.2 (39.2)	9.09 ± 1.28 (41.3)	41.4
Rock, uranium mineral	30.4	67.7 ± 4.1	56.0 ± 6.2 (71.8)	56.4 ± 6.3 (68.8)	14.5 ± 1.6 (65.9)	68.6
Soil	27.9	23.4 ± 1.9	19.0 ± 3.3 (24.2)	18.4 ± 1.1 (22.4)	4.82 ± 0.76 (21.9)	22.9
Chinese ceramic (cup)	43.7	48.6 ± 3.6	36.9 ± 3.3 (46.9)	39.6 ± 2.9 (48.3)	9.98 ± 0.74 (45.4)	47.3

Simple activity concentration analysis shows no activity equilibrium between each radionuclides–progenies of ^232^Th. Estimation and application of $\varepsilon_{red\;\left( x \right)}$—AC reduction factor in activity analysis of such radionuclides can give correct value. Activity concentration in the brackets are values calculated on the base $\varepsilon_{red\;\left( x \right)}$ of reference materials taken from Table 1.

## Conclusions

The ^232^Th radionuclide was measured in various different solid materials, including building materials and environmental samples. The described procedure makes it possible to quickly determine the activity concentration of ^232^Th in small solid (<50 g) samples. Using AC and single HPGe counting modes made it possible to verify the efficiency of coincidence signals. The FOM criterion was calculated in order to choose the best counting parameters. For the 583 keV line of ^208^Tl in single HPGe counting mode, the FOM criterion achieved the highest value. The AC mode can lead to a significant reduction of the count rate for ^208^Tl (583 keV), ^212^Bi (727 keV) and ^228^Ac (911 keV). For ^208^Tl reduction of counting rate in this mode has been significant. In AC mode average only 21 % of photons with energy 583 keV for ^208^Tl radionuclide in relation to single HPGe has been identified correctly. Not using external standards and precisely verifications the detection efficiency in AC mode seems to be useless or can lead to significant errors in assays radionuclide activity. Sophisticated electronic AC systems applied for activity concentration analysis of such radionuclides seems to be useless or at best can lead to significant mistakes in routine analysis.
